# Entinostat up-regulates the *CAMP* gene encoding LL-37 via activation of STAT3 and HIF-1α transcription factors

**DOI:** 10.1038/srep33274

**Published:** 2016-09-16

**Authors:** Erica Miraglia, Frank Nylén, Katarina Johansson, Elias Arnér, Marcus Cebula, Susan Farmand, Håkan Ottosson, Roger Strömberg, Gudmundur H. Gudmundsson, Birgitta Agerberth, Peter Bergman

**Affiliations:** 1 Department of Laboratory Medicine, Karolinska Institutet, Huddinge, Sweden; 2 Department of Medical Biochemistry and Biophysics, Karolinska Institutet, Stockholm, Sweden; 3 Institute of Environmental Medicine, Division of Biochemical Toxicology, Karolinska Institutet, Stockholm, Sweden; 4 Department of Microbiology, Tumor and Cell Biology, Karolinska Institutet, Stockholm, Sweden; 5 Department of Biosciences and Nutrition, Karolinska Institutet, Huddinge, Sweden; 6 Biomedical Center, University of Iceland, Reykjavík, Iceland

## Abstract

Bacterial resistance against classical antibiotics is a growing problem and the development of new antibiotics is limited. Thus, novel alternatives to antibiotics are warranted. Antimicrobial peptides (AMPs) are effector molecules of innate immunity that can be induced by several compounds, including vitamin D and phenyl-butyrate (PBA). Utilizing a luciferase based assay, we recently discovered that the histone deacetylase inhibitor Entinostat is a potent inducer of the CAMP gene encoding the human cathelicidin LL-37. Here we investigate a mechanism for the induction and also find that Entinostat up-regulates human β-defensin 1. Analysis of the *CAMP* promoter sequence revealed binding sites for the transcription factors STAT3 and HIF-1α. By using short hairpin RNA and selective inhibitors, we found that both transcription factors are involved in Entinostat-induced expression of LL-37. However, only HIF-1α was found to be recruited to the *CAMP* promoter, suggesting that Entinostat activates STAT3, which promotes transcription of *CAMP* by increasing the expression of HIF-1α. Finally, we provide *in vivo* relevance to our findings by showing that Entinostat-elicited LL-37 expression was impaired in macrophages from a patient with a STAT3-mutation. Combined, our findings support a role for STAT3 and HIF-1α in the regulation of LL-37 expression.

Innate immunity consists of a wide array of first line defences against invading pathogens. A major part of this defence system consists of antimicrobial peptides (AMPs). AMPs are evolutionary conserved and have been found in most living organisms^1^. In mammals there are two major classes of AMPs, the defensins (alpha, beta and theta) and the cathelicidins^2^,^3^, where LL-37 is the sole cathelicidin in humans and encoded by the *CAMP* gene. These peptides are synthesized at the host/microbe interface, e.g. epithelial linings and in certain immune cells^1^. AMPs exert microbicidal activity against bacteria, fungi, parasites and viruses, and can be considered as endogenous antibiotics^4^. Since they display overlapping specificity and different modes of action, the elimination of pathogens is very efficient and may be the reason why limited resistance has emerged against AMPs^5^. AMPs also have immune-modulatory activities in both the innate and the adaptive immune systems^6^,^7^,^8^. Dysregulation of AMP-expression has been linked to inflammatory disorders, such as psoriasis and Crohn’s disease, and infections like shigellosis and tuberculosis^9^,^10^,^11^,^12^.

We and others have shown that AMP expression can be induced by several small molecules^13^,^14^,^15^,^16^,^17^. One of the first identified inducers was butyrate, a short chain fatty acid that exhibits inhibitory effects towards histone deacetylases (HDAC). Butyrate was shown to induce cathelicidin expression in epithelial cells and also to clear bacterial infection in a rabbit model of shigellosis^13^,^18^. Furthermore, we have shown that several additional HDAC inhibitors also have the capacity to induce the expression of LL-37^19^. Interestingly, HDAC inhibition alone could not explain the induction of the *CAMP* gene, since the potency of HDAC inhibition did not correlate with the observed *CAMP* gene induction; hence the mechanism remains unresolved^19^.

We have previously developed a luciferase based screening assay in order to identify novel AMP-inducing compounds^19^. By using this assay we recently identified Entinostat and other related aroylated phenylendiamines (APDs) as potent inducers of LL-37, and that oral administration of Entinostat to a rabbit model of shigellosis clears the bacterial infection^20^. Entinostat is also known as a second generation HDAC inhibitor targeting class I HDACs and is currently being tested in clinical trials as an adjunctive therapy for various cancers^21^. It is known to act directly on tumour-cells, but may exert blocking capacity on immune-suppressor cells, such as T-regulatory cells and myeloid dendritic cells^22^,^23^,^24^. Entinostat is known to regulate the transcription factor Signal Transducer and Activator of Transcription 3 (STAT3)^23^,^25^, involved in the regulation of many genes related to immunity. Mutations in the gene encoding STAT3 cause autosomal-dominant hyper-IgE syndrome, a primary immunodeficiency characterized by recurrent staphylococcal infections, eczema as well as skeletal and connective tissue abnormalities^26^,^27^,^28^. Another transcription-factor related to AMP-expression is Hypoxia-inducible factor 1 (HIF-1), which is a master regulator of the cellular response to hypoxia. It has also been implicated as an immune modulator^29^,^30^ and shown to mediate the response to pathogens *in vivo* via regulation of AMPs^31^,^32^. HIF-1 is a dimer consisting of the inducible HIF-1α subunit, encoded by the gene *HIF1A,* and the constitutively expressed HIF-1β subunit^33^.

Given that we identified binding sites for STAT3 and HIF-1α in the promoter of LL-37 and that Entinostat is known to activate STAT3, we hypothesized that these transcription-factors were involved in Entinostat-mediated LL-37 transcription. Here we set out to test this hypothesis by using a combination of chemical inhibitors, short hairpin RNA-mediated knock-down of STAT3/HIF1-α expression and – finally – in macrophages from a STAT3-deficient patient.

## Results

### Entinostat induces the expression of the genes *CAMP* and *DEFB1* in HT-29 cells

**Figure 1:**
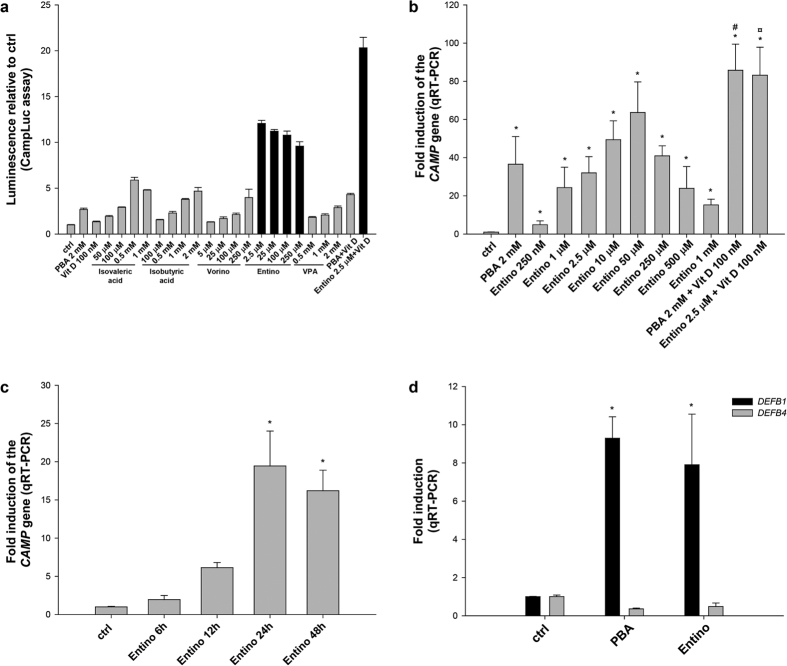
Induction of the *CAMP* gene by Entinostat in the Campluc reporter cells (**a**) and in HT-29 cells (**b**,**c**). **(a)** CampLuc reporter cells were stimulated for 24 h with the HDAC inhibitors phenylbutyrate (PBA, 2 mM), isovaleric acid (50 μM–1 mM), isobutyric acid (100 μM–2 mM), vorinostat (Vorino, 5–250 μM), Entinostat (Entino, 250–2.5 μM, black bars), and valproic acid (VPA, 0.5–2 mM). Vitamin D_3_ (Vit D, 100 nM) was also tested, alone or in combination with PBA (2 mM) or Entinostat (2.5 μM, black bar). The luciferase activity was assayed, and the results are expressed as luminescence relative to vehicle (ctrl). Graph is representative of at least 3 experiments (Compiled with data from Nylen *et al*.^19^). **(b)** Stimulation of HT-29 cells by Entinostat (250 nM-1 mM), PBA (2 mM) and PBA/Entinostat in combination with Vitamin D_3_ (Vit D, 100 nM) at different concentrations was determined for induction of the *CAMP* gene expression by qRT-PCR. Significantly altered expression is indicated vs ctrl: *p < 0.05; induction vs PBA: ^#^p < 0.05; induction vs Entinostat (Entino): ^¤^p < 0.05. **(c)** Treatment of HT-29 cells with Entinostat (2.5 μM) at different time-points (6–48 h) was analysed for the induction of *CAMP* gene expression by qRT-PCR. Significant altered expression vs ctrl is indicated: *p < 0.05. **(d)** The expression, of the genes *DEFB1* and *DEFB4* encoding HBD1 and HBD2, was assessed in HT-29 parental cells by qRT-PCR after stimulation with Entinostat (2.5 μM) or PBA (2 mM). Significantly altered expression vs ctrl is indicated: *p < 0.05.

Since the HDAC-inhibitors butyrate (BA) and phenylbutyrate (PBA) as well as their analogues isovaleric and isobutyric acids are known to induce *CAMP* gene expression^13^,^14^, we expanded on these findings and used the CampLuc reporter cell line^19^ to screen additional histone deacetylases (HDAC) inhibitors (e.g. valproic acid, Vorinostat, and other hydroxamic acids^19^) as well as Entinostat and related compounds^20^. Exposure to Entinostat caused a pronounced increase of proLL37-luciferase expression in the reporter cell line, significantly higher than other reported inducers^20^, here exemplified by comparison with Vorinostat and several short chain fatty acids (Fig. 1a). As previously observed with PBA, the combination of Entinostat with 100 nM of the active form of vitamin D_3,_ 1,25-dihydroxyvitamin D_3_ (Vit D), exhibited a synergistic effect, which was significantly more pronounced with Entinostat compared to PBA in the CampLuc assay (Fig. 1a and ref. 20). The ability of Entinostat to increase *CAMP* gene expression in the parental HT-29 cell line was confirmed by quantitative real-time PCR (qRT-PCR)^20^ where Entinostat causes a significant and concentration-dependent induction of the *CAMP* transcript at 24 h (250 nM to 1 mM) (Fig. 1b). The bell shaped curve is most likely explained by concentration-dependent cytotoxity (see Supplementary Fig. S1). The synergy observed with Entinostat and Vit D was confirmed in the parental cell-line on the mRNA level (Fig. 1b). For the following experiments we chose Entinostat at 2.5 μM, which caused a statistically significant induction on the mRNA level (in HT-29 cells) and on the protein level (in MN8CampLuc cells), but did not result in any cytotoxicity (compare Fig. 1a,b and Supplementary Fig. S1). At this concentration, Entinostat showed a time-dependent increase in *CAMP* gene expression in HT-29 cells, peaking at 24 h (Fig. 1c). Moreover, Entinostat also induced the transcription of the *DEFB1*gene, encoding the antimicrobial peptide beta-defensin-1 (HBD1), but not the gene *DEFB4* encoding HBD2, in HT-29 cells after 24 h stimulation (Fig. 1d).

### MAPK and NFκB pathways are not involved in Entinostat-mediated *CAMP* gene induction

Since the MAPK signalling pathway has been demonstrated to mediate the induction of LL-37 in colon epithelial cells^13^, intracellular signalling pathways, such as mitogen/extracellular signal protein kinase (MEK)-extracellular signal regulated protein kinase (ERK), p38 mitogen-activated protein kinase (MAPK) and c-Jun N-terminal kinases (JNK) were explored. CampLuc reporter cells were treated with U0126 or PD98059 (MEK1/2 inhibitors), SB203580 or SB202190 (p38 inhibitors) or SP600125 (JNK inhibitor). Notably, none of the inhibitors had any significant effects on Entinostat-mediated LL-37 induction (Supplementary Fig. S2).

The *CAMP* gene is induced by ER stress via a NF-κB-C/EBPα pathway in epithelial cells^34^,^35^. However, the NFκB inhibitor N-a-tosyl-L-phenylalanine chloromethyl ketone (TPCK) did not affect the increase of proLL-37-luciferase expression caused by Entinostat stimulation (Supplementary Fig. S2).

### Entinostat mediated induction of the *CAMP* gene is regulated by STAT3

**Figure 2:**
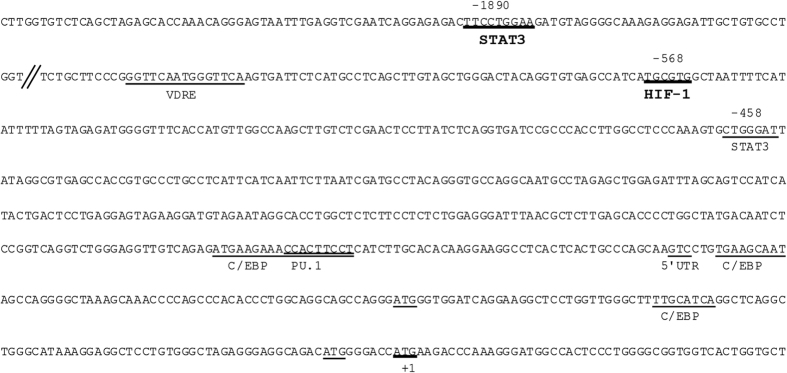
Analysis of transcription factor binding sites on the *CAMP* gene promoter. The *CAMP* gene promoter contains putative binding sites for the transcription factors STAT3 and HIF-1α as underlined in bold. Sequence analysis included 3000 bp proximal to the *CAMP* gene translational start site (+1) using the CONserved Regulatory Elements anchored Alignment (CONREAL) algorithm (http://conreal.niob.knaw.nl). Transcriptional start site is indicated as 5´UTR. Additional binding sites that are underlined are adopted from *Gombart et al.*^16^.

**Figure 3:**
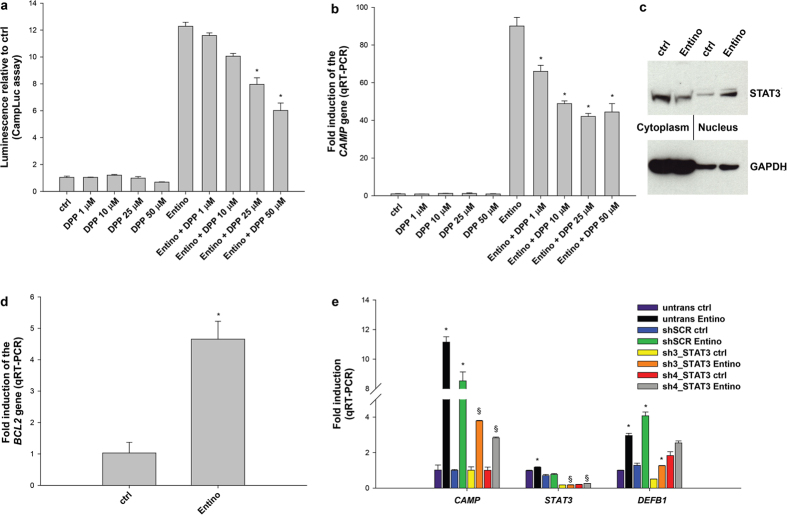
STAT3 mediates the *CAMP* gene induction by Entinostat. **(a)** The effect of the STAT3 inhibitor 5,15-diphenyl-21H,23H-porphine (DPP) at different concentrations (1–50 μM for 24 h) on Entinostat at 2.5 μM induction of the *CAMP* gene expression in the CampLuc reporter cells. **(b)** Transcriptional levels of the *CAMP* mRNA in the parental HT-29 cells after stimulation with 2.5 μM Entinostat (Entino), with or without DPP. Significant reduction vs Entinostat alone is indicated: *p < 0.05 (**a**,**b**). **(c)** Western blot analysis of subcellular fractions i.e. cytoplasm and nucleus, using anti-STAT3 upon induction with Entinostat (2.5 μM, 3 h) in HT-29 cells. GAPDH staining was utilized as a control for normalization. **(d)** The expression of the STAT3-responsive gene *BCL2* analysed by qRT-PCR in HT-29 cells stimulated for 24 h with 2.5 μM Entinostat or untreated (ctrl). Induction vs ctrl: *p < 0.05. **(e)** HEK293 cells were transfected with either a control vector (shSCR) or with shRNA specifically targeting STAT3 (sh3_STAT3 and sh4_STAT3) transcript. Cells were then stimulated with 2.5 μM Entinostat for 24 h or untreated (ctrl), and analysed for the expression of the genes *CAMP, STAT3*, and *DEFB1* (HBD1) by qRT-PCR, untransfected cells and untreated cells served as controls (ctrl). Significantly altered expression is indicated vs each respective ctrl: *p < 0.05; vs untransfected (untrans) cells stimulated with Entino: ^§^p < 0.05.

In order to determine which part of the regulatory region of the *CAMP* gene that might be involved in the Entinostat-mediated induction, *in silico* analysis of the *CAMP* promoter was performed. Sequence analysis using the ConREAL web-based algorithm for the identification of conserved transcription factor binding sites revealed a high-score binding site for STAT3 in position -1890. Additionally, Gombart *et al*. has previously described another STAT3 binding element around position -458 in the *CAMP* gene promoter^16^, where +1 ATG is the translation start site (numbers refer to previous mapping of the gene^36^, Fig. 2). To confirm that STAT3 was involved in Entinostat induced *CAMP* gene transcription, we employed 5,15-diphenyl-21H,23H-porphine (DPP), a selective STAT3 inhibitor. DPP could reduce, but not completely block, the effects of Entinostat, both on the protein level (measured in CampLuc cells, Fig. 3a) and on the mRNA level (in HT-29 cells, Fig. 3b) in a concentration-dependent manner (1 μM to 50 μM).

By performing fractionation of HT-29 cell lysates we found that Entinostat induced translocation of STAT3 from the cytoplasm to the nucleus after 3 h stimulation (Fig. 3c).

To further confirm STAT3 activation by Entinostat, we analysed the expression of a known STAT3 downstream target gene, *BCL2*, by qRT-PCR. As shown in Fig. 3d, *BCL2* levels were significantly increased upon Entinostat stimulation.

Next, we examined the effect of the knock-down of STAT3 on the *CAMP* gene expression. As shown in Fig. 3e, STAT3 silencing by the two shRNA vectors sh3_STAT3 and sh4_STAT3 significantly decreased the Entinostat-elicited *CAMP* gene induction. The *DEFB1* gene expression was down-regulated by sh3_STAT3, but not by sh4_STAT3. In order to study a possible recruitment of STAT3 to the putative binding sites in the promoter region of the *CAMP* gene, chromatin immunoprecipitation (ChIP) experiments were performed on HT-29 cells stimulated with either vehicle or Entinostat. However, no enriched binding of STAT3 to either binding site in the *CAMP* gene promoter was detected upon 3 h stimulation with Entinostat (Data not shown).

### *De novo* protein synthesis is required for full inducing effect by Entinostat

**Figure 4:**
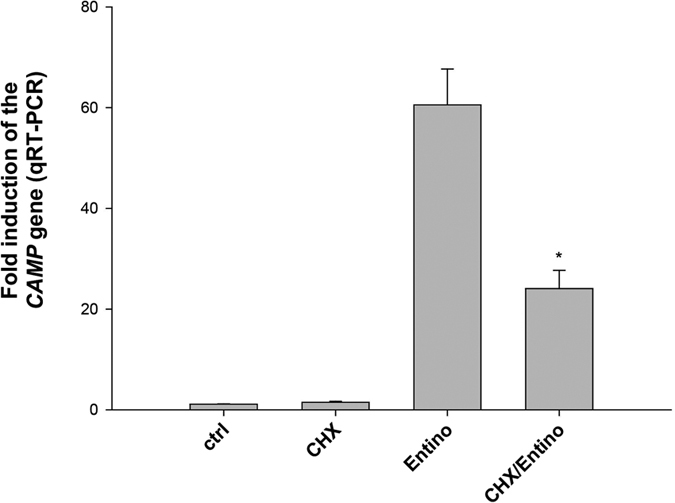
Induction of the *CAMP* gene is partly dependent on *de novo* protein synthesis. *De novo* protein synthesis in HT-29 cells was inhibited by incubation with 1 μg/ml cycloheximide (CHX) in the presence or absence of 2.5 μM Entinostat for 24 h. The levels of the *CAMP* transcript were measured by qRT-PCR. CHX significantly reduced Entinostat-mediated induction of the *CAMP* gene *p < 0.05.

We previously reported that the mechanism of PBA-induced *CAMP* gene involves *de novo* protein synthesis^14^. To investigate whether this was also the case for Entinostat, we employed the protein synthesis inhibitor cycloheximide (CHX) in HT-29 cells. At 1 μg/ml of CHX, the Entinostat induced expression of the *CAMP* gene was significantly attenuated (by 50–60%), suggesting a secondary effect of this compound on the transcription of the *CAMP* gene (Fig. 4). As a control experiment, CHX in combination with Vit D was analysed, and the induction of *CYP24*, a known Vit D responsive gene, which is independent of *de novo* protein synthesis, was assessed by qRT-PCR (Supplementary Fig. S3). A potent increase of *CYP24*-gene expression was observed, despite treatment with 1 μg/ml of CHX, ruling out the chance of a non-specific down-regulation of gene expression.

### HIF-1α expression and activation is required for *CAMP* gene induction by Entinostat

**Figure 5:**
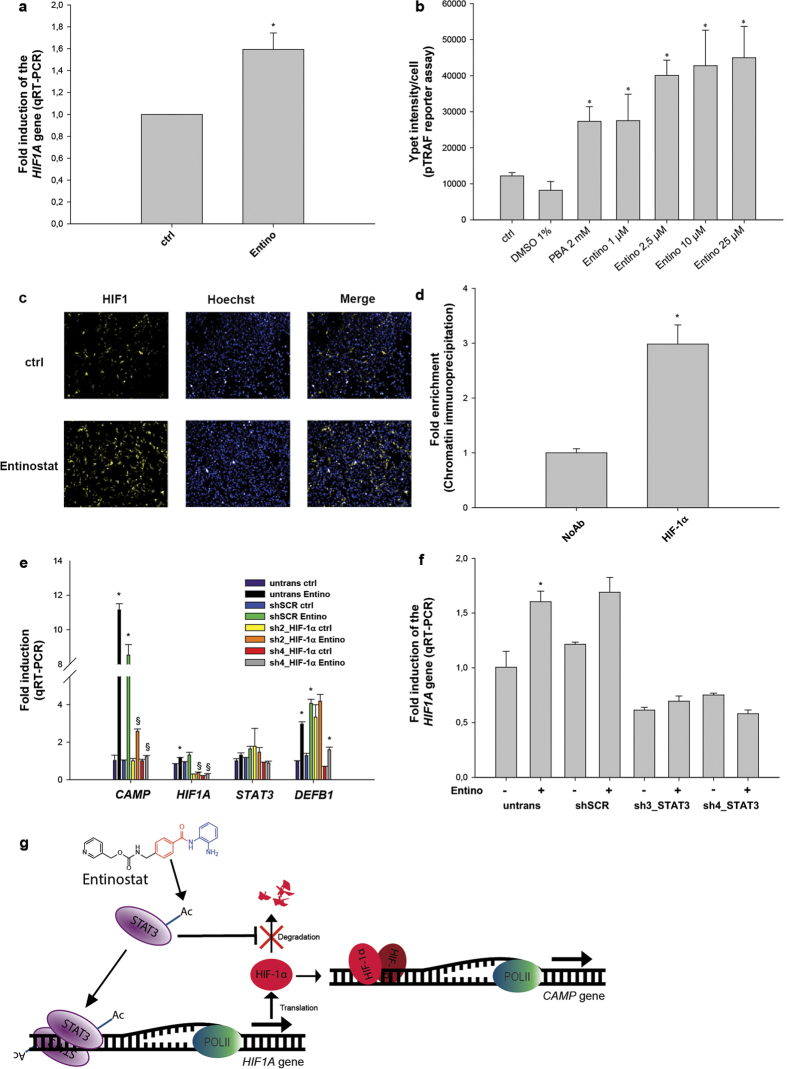
*CAMP* gene induction by Entinostat requires HIF-1α synthesis and activation. **(a)** HIF-1α mRNA levels were analysed by qRT-PCR in HT-29 cells upon Entinostat (Entino) stimulation (2.5 μM, 24 h). Induced expression vs ctrl: *p < 0.05. **(b,c)** HEK293 cells transfected with the reporter vector pTRAF and stimulated for 24 h **(b)** with either 2 mM PBA or Entinostat (1–25 μM). HIF-1α activation was measured as Ypet intensity/cell. **(c)** Ypet fluorescence as seen in representative fields of unstimulated (ctrl) and 2.5 μM Entinostat (Entino) stimulated HEK293 cells. **(d)** ChIP assay for HIF-1α recruitment to the *CAMP* gene promoter was performed in HT-29 cells stimulated for 3 h with or without Entinostat. Significance vs ctrl: *p < 0.05. **(e)** HIF-1α knock-down was performed in HEK293 cells by transfecting with either a control vector (shSCR) or with shRNA specifically targeting HIF-1α (sh3_ HIF-1α and sh4_ HIF-1α) transcript. Cells were then stimulated with or without 2.5 μM Entinostat for 24 h, and analysed for the expression of the genes *CAMP, HIF1A, STAT3*, and *DEFB1* (HBD1) expression by qRT-PCR. Significance vs each respective ctrl: *p < 0.05; significance vs untransfected (untrans) cells stimulated with Entino: ^§^p < 0.05. **(f)** HEK293 cells were transfected with either a control vector (shSCR) or with shRNA specifically targeting STAT3 (sh3_STAT3 and sh4_STAT3) transcript. Cells were then stimulated with or without 2.5 μM Entinostat for 24 h, and then analysed for HIF-1α expression by qRT-PCR. Significance vs untransfected (untrans) control (ctrl) cells: *p < 0.05. (**g**) Representation of a proposed mechanism where Entinostat activates STAT3, possibly by inhibition of deacetylation of the transcription factor, which in turn increase expression of the *HIF1A* gene and stabilizes the protein product HIF-1α. Induced *CAMP* gene expression is activated by direct binding of HIF-1α.

Since the expression of *CRAMP*, the mouse cathelicidin, depends on HIF-1α^31^, we postulated that Entinostat could regulate the human *CAMP* gene via this transcription factor. As shown in Fig. 5a, stimulation with Entinostat caused an up-regulation of *HIF1A* expression on the mRNA level.

Next, we addressed whether increased *HIF1A* mRNA correlated to enhanced downstream activity. A reporter vector named pTRAF^37^, which allows the imaging of HIF-1α activation via the yellow fluorescent protein YPet in HEK293 cells, was utilized. We found that both PBA (2 mM) and Entinostat (1–25 μM) caused a robust activation of HIF-1α (Fig. 5b). In Fig. 5c, one representative field of cells stimulated with Entinostat compared to cells incubated with vehicle alone is shown.

*In silico* analysis of the *CAMP* promoter (see Fig. 2) revealed one high-score binding site for HIF-1α in position -568 (where +1ATG is the translation start site). Therefore, ChIP analysis was performed on HT-29 cells to assess the recruitment of HIF-1α to the *CAMP* gene promoter. As shown in Fig. 5d, the proximal region of the *CAMP* promoter showed a significant enrichment of bound HIF-1α. As a proof of concept, *HIF1A* gene expression was down regulated using shRNA, which prevented Entinostat-induced *CAMP* gene expression (by ~80–90%) (Fig. 5e). Interestingly, STAT3 knock-down with shRNA constructs resulted in a down-regulation of HIF-1α and completely prevented Entinostat-induced increase of *HIF1A* mRNA (Fig. 5f). STAT3 mRNA levels were not significantly affected by HIF-1α silencing, whereas the two vectors used (sh2_HIF-1α and sh4_HIF-1α) had opposing effects on *DEFB1* gene expression (Fig. 5e). These results implicate that regulation of the CAMP gene in human macrophages, at least in part, involves STAT3 that in turn can regulate HIF1α (Fig. 5g).

### *In vivo* relevance of the STAT3-mediated pathway in the regulation of the *CAMP* gene

**Figure 6:**
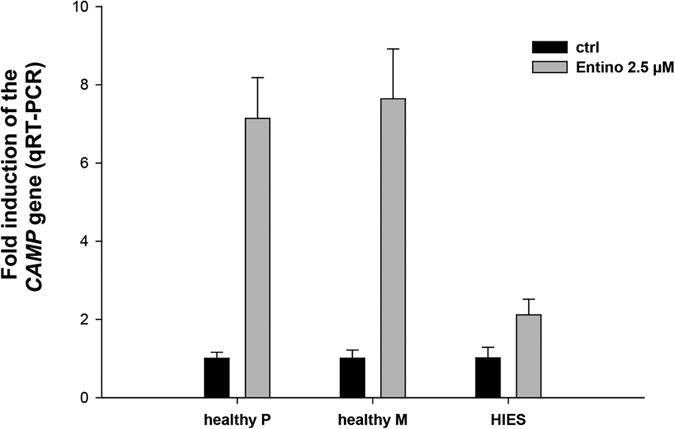
*CAMP* induction by Entinostat was reduced in macrophages from a hyper-IgE (HIES)-patient. Macrophages obtained from a HIES patient and from two healthy controls (healthy P and healthy M) were stimulated (grey bars) or untreated (black bars) with Entinostat (2.5 μM, 24 h) and then assayed for *CAMP* gene induction by qRT-PCR.

Next, the *in vivo* relevance of STAT3 in the regulation of the *CAMP* gene was investigated in immune cells from one patient with hyper-IgE syndrome (HIES). HIES patients bear dominant-negative STAT3 mutations leading to reduced STAT3 signalling, and they suffer from recurrent staphylococcal and candida infections, pneumonia and eczema^38^. We previously found that HIES patients have an impaired release of AMPs in nasal fluid in response to pathogens^39^. Therefore we analysed the response to Entinostat, in terms of *CAMP* gene induction, in HIES macrophages compared to macrophages from healthy controls. Interestingly, Entinostat induced the *CAMP* gene in healthy control macrophages, but hardly at all in HIES macrophages (Fig. 6), suggesting a crucial role of STAT3 in the regulation of the *CAMP* gene in human macrophages.

## Discussion

The HDAC inhibitor Entinostat is a potent inducer of the human antimicrobial peptide LL-37 expression^20^, as shown in several cell types, and it works in synergy with the active form of vitamin D. Here, we dissected the molecular basis of this induction and unravelled a complex interplay between the transcription factors STAT3 and HIF-1α in the regulation of the *CAMP-*gene encoding LL-37. Our data suggest a model where Entinostat activates STAT3, which subsequently leads to activation of HIF-1α and downstream transcription of the *CAMP* gene.

HDAC inhibitors have been reported to induce AMP-expression. For example, the non-selective HDAC inhibitors trichostatin A and sodium butyrate up-regulate the expression of LL-37 in human airway epithelial-40, in gastric-, hepatocellular-41 and colon epithelial- cells^13^. The HDAC inhibitor phenylbutyrate (PBA) has previously been shown to up-regulate LL-37 expression in lung epithelial cells and in monocytes^14^. In our luciferase reporter assay Entinostat dramatically induced the expression of LL-37^20^. This induction was substantially higher than with other more potent HDAC inhibitors and at a much lower concentration than the positive control PBA^20^. Histone acetylation plays a critical role in the regulation of gene transcription by causing chromatin remodelling and allowing the binding of transcription factors to regulatory DNA-elements^42^. The molecular basis of *CAMP* induction by PBA is, however, more complex and partly mediated via the vitamin D receptor VDR^43^, but may also depend on *de novo* protein synthesis^14^. Entinostat - like PBA - is known to be an HDAC inhibitor and thus regulates the expression of many genes. However, we have not observed any clear correlation between documented potency of HDAC inhibition and induction of LL-37^19^,^20^. Given that Entinostat and several other related compounds was superior to all other HDAC-inhibitors analysed^19^, we hypothesized that additional mechanisms, apart from HDAC inhibition, are involved in Entinostat-mediated regulation of the *CAMP* gene.

Entinostat also enhanced the transcription of the *DEFB1* gene encoding human β-defensin 1 (HBD1), an important peptide of innate defences at epithelial surfaces^44^. However, the *DEFB4* gene encoding β-defensin 2 (HBD2) was not affected by Entinostat stimulation. It is plausible that the expression of additional AMPs can be modulated by Entinostat. This possibility is quite appealing, as the induction of an array of AMPs is favourable for the host during infection. Interestingly, AMPs are often co-regulated^5^,^45^ and it is also known that LL-37 is downregulated by several pathogenic bacteria^18^,^46^. We therefore consider LL-37 as a marker for a healthy epithelial barrier and as a representative for innate effectors. The combination of Entinostat with vitamin D results in a synergistic up-regulation of LL-37. The molecular mechanism behind this synergy has yet to be elucidated. For Entinostat alone, however, Shen *et al*.^23^ showed increased acetylation of STAT3 upon Entinostat challenge. STAT3 in turn is linked to host defence and inflammation, and *in silico* analyses indicate the presence of STAT3 responsive elements in the promoter of the *CAMP* gene. Therefore we hypothesized that STAT3 could mediate Entinostat-elicited induction of LL-37 expression in our model. Indeed, blocking STAT3 signalling pathways with either a pharmacological inhibitor or with RNA-silencing strategies reduced LL-37 induction by Entinostat, providing evidence for a critical role of this transcription factor in the induced expression of the *CAMP* gene. Entinostat activated STAT3 signalling in HT-29 cells, as shown by the enhanced transcription of *BCL2*, a known STAT3 downstream target gene^47^. We also observed a translocation of STAT3 from the cytoplasm to the nucleus, suggesting that upon Entinostat stimulation, STAT3 is accumulated in the nuclear compartment, where it can bind and activate target genes. In a murine model of infection with *Citrobacter rodentium*, a pathogen that mimics *Escherichia coli* infection in humans, the expression of AMPs, such as RegIIIγ and Pla2g2a is dependent on STAT3 activation in the intestine. Furthermore, STAT3 deletion causes increased susceptibility to *Citrobacter rodentium* infection, with higher bacterial load, severe gut inflammation and dissemination of bacteria to distant organs^48^. Interestingly, we could not detect recruitment of STAT3 to the *CAMP* promoter, suggesting an indirect role for this transcription factor in the regulation of the *CAMP* gene. Further, we observed an induction of the gene *HIF1A* (encoding HIF-1α) after treatment with Entinostat. HIF-1α is a master regulator of the homeostatic response to hypoxia and activates the transcription of many target genes^33^. HIF-1α activation classically occurs via hypoxia-induced stabilization of the HIF-1α subunit. However, oxygen-independent induction of HIF-1α expression has been documented. Lipopolysaccharide causes HIF-1α accumulation in macrophages through transcriptional and translational activation, in an hypoxia-independent fashion^49^. NFκB, a key regulator of the immune response to infections, was found to mediate bacteria-elicited increase of HIF-1α mRNA in macrophages^50^. Tumour necrosis factor-α (TNF-α), another crucial inflammatory mediator, induced HIF-1α expression in macrophages under normoxia^51^, providing another link between inflammation and HIF-1α stabilization in immune cells. T-cell receptor ligation enhanced HIF-1α expression, especially in the pro-inflammatory Th17 cells, via a STAT3 dependent mechanism^52^. STAT3 also mediates IL-6 and TGF-β elicited induction of HIF-1α mRNA^53^. Here we did not observe an involvement of NFκB in the inducing effect of Entinostat on the *CAMP* gene expression (Supplementary Fig. S2). On the other hand, STAT3 silencing completely abrogated the up-regulation of HIF-1α mRNA caused by Entinostat, providing evidence for a critical role of STAT3 in the transcriptional regulation of the *HIF1A* gene.

Next, we asked whether increased expression of HIF-1α was reflected by a functional activation of this transcription factor. The results obtained with the pTRAF reporter system clearly demonstrated an activation of HIF-1α by Entinostat, which was detected as increased production of the corresponding fluorescent protein downstream of a HIF-1α binding element in the promoter region of the reporter plasmid. Interestingly, ChIP analyses showed a recruitment of HIF-1α to the *CAMP* promoter in the proximity of a HIF-1α binding site upon Entinostat stimulation. Thus, the next question to address was whether HIF-1α was needed for LL-37 induction by Entinostat. By using HIF-1α-targeting shRNA we demonstrated that the knock down of this transcription factor significantly reduced the inducing effect of Entinostat on LL-37 expression. We did not observe a clear effect of HIF-1α silencing on *DEFB1* gene expression, suggesting a different mechanism for the induction of this gene by Entinostat. Notably, HIF-1α knock down did not affect STAT3 mRNA levels, indicating that in the interplay between these two transcription factors, STAT3 is not transcriptionally regulated by HIF-1α.

The current results are in line with previous reports, suggesting that HIF-1α plays a key role in host innate immunity. HIF-1α deficient macrophages displayed reduced migration and bacterial clearance^29^, and reduced bacterial phagocytosis^30^. Mice lacking myeloid HIF-1α showed higher susceptibility to infection with *Streptococcus pyogenes* and decreased neutrophil production of granule proteases and the murine cathelicidin peptide CRAMP^31^. Conversely, activation of HIF-1α supported myeloid cell production of host defence factors and improved bactericidal activity^31^. In addition, pharmacological stabilization of HIF-1α enhanced phagocyte-mediated clearance of methicillin-resistant *Staphylococcus aureus*^54^. Moreover, our results indicate that STAT3 is involved in the regulation of HIF-1α, since silencing of STAT3 by shRNA abrogates Entinostat-mediated induction of HIF-1α mRNA.

To further corroborate an *in vivo* role for STAT3 in AMP-expression, we obtained cells from a patient with hyper-IgE syndrome (HIES). Interestingly, Entinostat gave little induction of LL-37 in macrophages obtained from this HIES patient, with an impaired STAT3 signalling, compared to control macrophages from two healthy controls. Furthermore, we have previously demonstrated that HIES patients have an impaired release of AMPs in nasal fluid in response to pathogenic bacteria^39^. Therefore our results implicate that STAT3 is involved in the regulation of the *CAMP* gene in human macrophages.

## Materials and Methods

### Ethical statement

Written informed consent was obtained from the HIES patient and healthy controls, who were recruited from the Immunodeficiency Unit, Karolinska University Hospital, Stockholm, Sweden. The study was approved by the regional ethical committee in Stockholm (dnr 2011/116-31/4) and all experiments were performed in accordance with the approved guidelines at Karolinska Institutet, Stockholm, Sweden.

### Nomenclature of the transcription factor HIF-1

The HIF-1 transcription factor consists of an α- and a β- subunit. Herein we use primers, shRNA and antibodies raised against the inducible HIF-1α subunit. Throughout the text we therefore use HIF-1α for describing both the dimer and the α-subunit. *HIF1A* is the name of the gene encoding HIF-1α.

### Cell culture

The human colonic epithelial cell line HT-29 was obtained from the American Type Culture Collection: HTB-38 (Rockville, Md., USA) and was cultured in RPMI 1640 (Gibco, Carlsbad, CA, USA), supplemented with 10% Fetal Calf Serum (FCS; Gibco), 100 μg/ml streptomycin and 100 U/ml penicillin (Invitrogen, Carlsbad, CA, USA), in a 5% carbon dioxide atmosphere at 37 °C. HT-29 cells stably transfected with a plasmid containing the *CAMP* gene ( henceforth termed MN8CampLuc cells), including the upstream promoter region of approximately 3000 base-pairs (bp) fused with the firefly luciferase gene^19^, were grown under the same conditions as the parental HT-29 cells^19^. The human embryonic kidney 293 cell line (HEK293) was cultured in Dulbecco’s modified Eagle’s medium (DMEM; Gibco) and Eagle’s Minimum Essential Medium (MEM) (ATCC, Manassas, Virginia, USA) supplemented with 10% FBS (GE Healthcare) as well as 100 U/ml penicillin and 100 μg/ml streptomycin (Biochrom, Berlin, Germany) in a 5% carbon dioxide atmosphere at 37 °C.

Mononuclear cells were isolated from human peripheral blood of healthy volunteers or a hyper-IgE (HIES) patient by density gradient centrifugation with Ficoll-Paque Plus (GE Healthcare, Little Chalfont, UK) and then seeded into 6-well plates in serum free RPMI. After 2 hours, medium was replaced with fresh RPMI containing 10% FCS supplemented with 50 ng/ml macrophage colony-stimulating factor (M-CSF) for differentiation to the M2 macrophage subset. After 7 days, cells were stimulated for 24 h with Entinostat for qRT-PCR experiments.

### Quantitative real-time polymerase chain reaction (qRT-PCR)

For evaluation of induction on mRNA level, cells were seeded in 6-well plates and grown for 24 h before incubation with stimuli or vehicle for different time points. Total RNA was extracted using the ISOLATE II RNA Mini Kit (Bioline USA Inc., Taunton, MA, USA). cDNA was synthesized from 1 μg of RNA using iScript cDNA Synth RT-PCR kit according to the user manual (Bio-Rad, Hercules, CA, USA). The resulting cDNA was then amplified in technical duplicates by quantitative real-time PCR (qRT-PCR) using iQ SYBR Green (Bio-Rad) in the CFX96 Real-Time PCR Detection System (Bio-Rad). The relevant transcripts were analysed by the 2^−∆∆Ct^ method in relation to transcripts of the housekeeping gene 18S. Primers used for amplifying the *CAMP* transcript were 5′-TCACCAGAGGATTGTGACTTCAA-3′ (forward (fw)) and 5′-TGAGGGTCACTGTCCCCATAC-3′ (reverse (rev)); for *STAT3* 5′-GGAGGAGTTGCAGCAAAAG-3′ (fw) and 5′-TGTGTTTGTGCCCAGAATGT-3′ (rev); for *HIF1A* 5′-CCATTAGAAAGCAGTTCCGC-3′ (fw) and 5′-TGGGTAGGAGATGGAGATGC-3′ (rev); for *DEFB1* 5′-ATGGCCTCAGGTGGTAACTTTC-3′(fw) and 5′-CACTTGGCCTTCCCTCTGTAAC-3′(rev); for *DEFB4* 5′-GCCTCTTCCAGGTGTTTTTG-3′ (fw) and 5′-GAGACCACAGGTGCCAATTT-3′ (rev); for *BCL2* 5′-AGATGTCCAGGCAGCTGCACCTGAC-3′ (fw) and 5′-ATAGGCACCCAGGGTGATGCAAGCT-3′ (rev); for *18S* 5′-GTAACCCGTTGAACCCCATT-3′ (fw) and 5′-CCATCCAATCGGTAGTAGCG-3′ (rev).

### Luciferase activity assay

MN8CampLuc cells were seeded into 96-well plates in duplicates at a density of 6 × 10^4^ cells/well and cultured for 48 h in RPMI growth medium. Cells were then exposed to either vehicle (control) or test compounds for 24 h. Isovaleric acid, Isobutyric acid and valproic acid were obtained from Sigma-Aldrich. Entinostat and Vorinostat were purchased from LC laboratories (Woburn, MA, USA). Following treatment, cells were harvested and lysates were assayed for luciferase activity using the Luciferase assay kit (Promega, Madison, WI, USA) in accordance to manufacturer’s instructions. Luminescence was recorded using an Infinite M200 microplate reader (TECAN, Infinite, Männedorf, Switzerland).

U0126, PD98059 (MEK1/2 inhibitors), SB203580, SB202190 (p38 inhibitors) and SP600125 (JNK inhibitor) were all purchased from Calbiochem (Nottingham, UK) and used alone or in combination with 2.5 μM Entinostat.

### Cell lysate fractionation

HT-29 cells were seeded in 10 cm dishes and treated with or without 2.5 μM Entinostat for 3 h. Cells were then harvested in ice-cold phosphate-buffered saline (PBS) containing 1 mM EDTA and centrifuged at 3000 rpm for 5 min at 4 °C. Then cells were resuspended in cold harvest buffer (10 mM HEPES pH 7.9, 50 mM NaCl, 0.5 M sucrose, 0.1 mM EDTA, 0.5% Triton X-100, 1 mM Dithiothreitol (DTT), 10 mM tetrasodium pyrophosphate, 100 mM sodium fluoride, 17.5 mM beta-glycerophosphate, 1 mM phenylmethylsulphonyl fluoride (PMSF), supplemented with Complete Protease Inhibitor Cocktail tablets (Roche, Indianapolis, IN, USA) and centrifuged at 1000 rpm for 10 min at 4 °C. The supernatants, containing membrane and cytoplasmic proteins, were centrifuged at 14000 rpm for 15 min. The pellets, containing the nuclei, were resuspended in buffer A (10 mM HEPES pH 7.9, 10 mM KCl, 0.1 mM EDTA, 0.1 mM EGTA, 1 mM DTT, 1 mM PMSF, supplemented with Complete Protease Inhibitor Cocktail tablets). Afterwards, nuclei were centrifuged at 1000 rpm for 10 min and pellets were lysed in buffer B (10 mM HEPES pH 7.9, 500 mM NaCl, 0.1 mM EDTA, 0.1 mM EGTA, 0.1% nonyl phenoxypolyethoxylethanol (NP) -40, 1 mM DTT, 1 mM PMSF, supplemented with Complete Protease Inhibitor Cocktail tablets) on a high-speed vortex at 4 °C. After centrifugation at 14000 rpm for 10 min, supernatants containing nuclear extracts were collected. Both cytoplasmic and nuclear fractions were incubated with lithium dodecyl sulfate (LDS) buffer containing 50 mM DTT for 5 min at 95 °C. Samples were then subjected to gel electrophoresis and Western blot analysis. Membranes were stained for STAT3 and GAPDH using antibodies purchased from Santa Cruz Biotechnology (Santa Cruz, CA, USA).

### Chromatin immunoprecipitation (ChIP)

HT-29 cells were grown to 80% confluency in 10 cm culture dishes, treated with 2.5 μM Entinostat or control and harvested 3 h post treatment. Cells were then cross-linked utilizing 1% formaldehyde for 10 min at room temperature with gentle agitation. Cross-linking reaction was stopped by the addition of glycine to a final concentration of 0.125 M for 5 min at RT. Cells were washed with cold PBS, harvested, and resuspended in 400 μl lysis buffer (50 mM HEPES pH 8.0, 1 mM EDTA, 0.5 mM EGTA, 140 mM NaCl, 10% glycerol, 0.5% NP-40, 0.25% Triton X-100, and 1 mM PMSF, supplemented with Complete Protease Inhibitor Cocktail tablets) and sonicated by the Bioruptor Plus (Model UCD-300, Diagenode, Liège, Belgium) for 5 cycles of 30 s with a 30 s rest period between pulses. The chromatin-containing supernatants obtained after centrifugation were incubated with 1μg of antibodies specific for STAT3 (Santa Cruz Biotechnology) or HIF-1α (BD Biosciences, Franklin Lakes, NJ, USA) overnight at 4 °C. The following day the supernatants were incubated with 50μl protein A/G Sepharose (50% slurry; Santa Cruz Biotechnology) under gentle agitation for 2 h at 4 °C. The pellets were then washed twice with 1ml washing buffer (10 mM Tris–HCl pH 8.0, 1 mM EDTA, 0.5 mM EGTA, 200 mM NaCl, and 1 mM PMSF), three times with washing buffer containing an increased salt concentration (500 mM NaCl), and once again with ordinary washing buffer. After washing, the pellet was resuspended in 110μl Tris-EDTA (TE) buffer (10 mM Tris–HCl pH 8.0 and 1 mM EDTA) with 1% SDS and the cross-links were reversed by overnight incubation at 66 °C. Using a PCR purification kit (Promega), DNA was isolated and eluted in 50μl elution buffer. Immunoprecipitated samples and total input were analysed by PCR using two sets of primers: ChIP1 primers, designed to amplify a 324 bp region of the *CAMP* gene promoter, were used to assess recruitment of HIF-1α and STAT3 transcription factors proximal to the *CAMP* gene. ChIP1 primers were; 5′-GCCACCGTGCCCTGCCTCATTCATCAATTC-3′(fw), −440 from +1ATG; 5′-GGGTGTGGGCTGGGGTTTGCTTTA-3′ (rev), −116 from +1 ATG). ChIP2 primers, designed to amplify a 207 bp region of the *CAMP* promoter, were used to study the distal STAT3 binding site; 5′- AGCTAGAGCACCAAACAGGG-3′(fw), -1939 from +1 ATG ; 5′- CACGTATGCCCCCATCACAT-3′ (rev), −1732 from +1 ATG) (see Fig. 2).

### HIF-1α activation with the pTRAF reporter vector

Activation of HIF-1α was studied using the pTRAF (plasmid for transcription factor reporter activation based on fluorescence) reporter plasmid according to Johansson *et al*.^37^. Activation of HIF-1α promotes the expression of YPet by binding to a suitable response element, guiding the expression of this fluorescent protein. The fluorescence can be quantified and is directly correlated to the degree of HIF-1α activation.

Briefly, HEK293 cells were seeded at a density of 18000 cells/well in 96-well plates (Biocoat Collagen I plate) approximately 24 h before transfection with 0.05 μg DNA, 5 μl OptiMEM and 0.1 μl TurboFect (Invitrogen) diluted in 55 μl medium per well. The DNA mixture (DNA, OptiMEM and TurboFect) was first incubated for 20 min, before the complete medium was added. After approximately 20 h of transfection cells were exposed to Entinostat at different concentrations for 24 h. To prepare the samples for analysis, cells were treated with 40 ng/ml Hoechst for 30 min to stain the nuclei and subsequently fixed in 2% ice-cold paraformaldehyde for 10 min in RT. The fixed cells were covered with PBS and fluorescence was measured using the Operetta^®^ High Content Imaging System (PerkinElmer). For single cell quantification, seven fields for each cell culture well, covering edges and centre, were recorded in two channels for fluorescence detection of HIF-1α (Ypet) (excitation: 490–510 nm; emission: 520–560 nm) and Hoechst (excitation: 360–400 nm; emission: 410–480 nm). The exposure times were fixed for each channel, with all samples analysed in the same settings. Determinations of fluorescence signals were subsequently performed using the Columbus (PerkinElmer Waltham, MA, USA,) and Excel (Microsoft Redmond, WA, USA) computer programs. Briefly, individual cells were identified based upon the Hoechst staining and the corresponding cytosols were defined using the fluorescent signal that accorded to HIF-1α expression. The intensities of these signals were quantified as total signals within the defined cell area. The resulting single-cell results were subsequently exported from the Columbus software and further analysed using the Excel and GraphPad Prism (GraphPad Software, San Diego, CA, USA) computer programs. To determine accumulated responses on cell culture-level, all single-cell signals within an experiment were summarized and corrected for total cell numbers. For more detailed information, see Johansson *et al*.^37^.

### STAT3 and HIF-1α knock-down experiments

HEK293 cells were seeded in 24-well plates and grown to 80% confluency. Then the cells were transiently transfected with 1 μg of either a scramble vector (shSCR) or with shRNA vectors specifically targeting STAT3 transcript (sh3_STAT3, TRCN0000329886 and sh4_STAT3, TRCN0000020843) or HIF-1α transcript (sh2_ HIF-1α, TRCN0000003809 and sh4_HIF-1α, TRCN0000003811) (all plasmids were obtained from Sigma-Aldrich). The vectors and 2 μl of Turbofect were diluted in OptiMEM to a final volume of 100 μl. Twenty-four hours post-transfection, the medium was replaced and cells were treated with either vehicle or Entinostat for an additional 24 h. The following day, cells were harvested and assayed by qRT-PCR as described above.

## Additional Information

**How to cite this article**: Miraglia, E. *et al*. Entinostat up-regulates the *CAMP* gene encoding LL-37 via activation of STAT3 and HIF-1α transcription factors. *Sci. Rep.* **6**, 33274; doi: 10.1038/srep33274 (2016).

## Supplementary Material

Supplementary Information
