# Supplementary material for: Entinostat up-regulates the CAMP gene encoding LL-37 via activation of STAT3 and HIF-1α transcription factors
Source: Sci Rep. 2016 Sep 16;6:33274. doi: 10.1038/srep33274 (PMC5025742; doi:10.1038/srep33274)
Supplement: Supplementary Information [file srep33274-s1.pdf]

Entinostat up-regulates the *CAMP* gene encoding LL-37 via activation of STAT3 and HIF-1α transcription factors

Erica Miraglia, Frank Nylén, Katarina Johansson, Elias Arnér, Marcus Cebula, Susan Farmand,

Håkan Ottosson, Roger Strömberg, Gudmundur H. Gudmundsson,

Birgitta Agerberth, Peter Bergman

Supplementary Figure S1

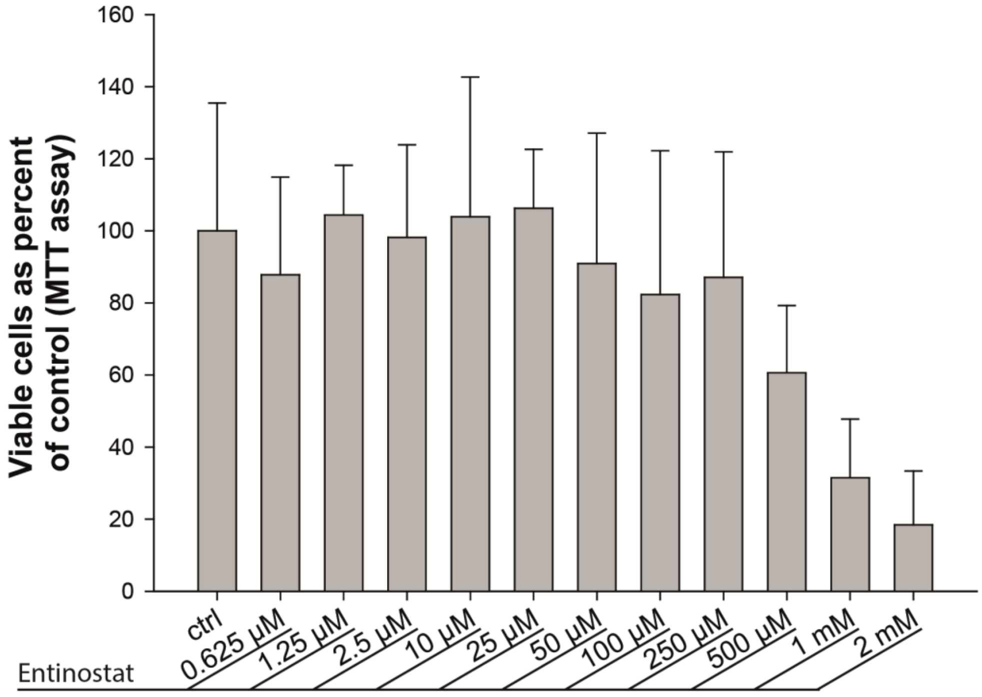

Supplementary Figure S1. Concentration dependent cytotoxicity elicited by entinostat. Cytotoxicity data from the MTT assay performed in technical duplicates on HT-29 cells; n=2 for 250-2.5 µM and control, n=1 for the other concentrations. Cells were stimulated for 24 h with entinostat at the concentrations depicted in the graph. (3-(4,5-dimethylthiazol-2-yl)-2,5-diphenyltetrazolium bromide (MTT)).

Supplementary Figure S2

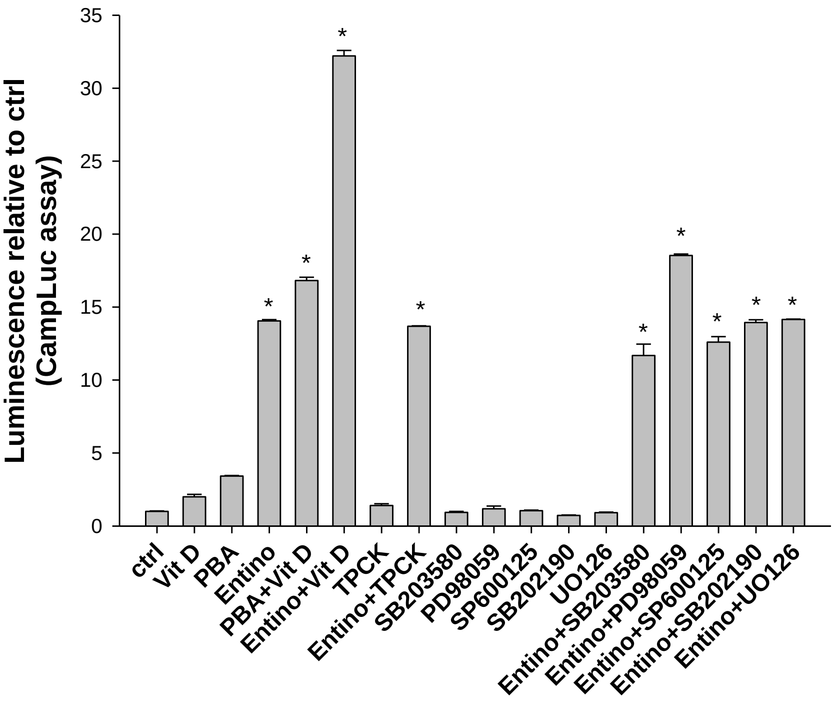

Supplementary Figure S2. Entinostat is not regulating the *CAMP* gene via NFκB, MEK1/2, p38 or JNK pathway. CampLuc reporter cells were stimulated for 24 h with negative (vehicle ctrl) and positive (Vit D 100 nM, PBA 2 mM, entino 2.5µM, PBA + Vit D and entino + Vit D) controls. Inhibitors for different signalling pathways were added separately to the cells at 10 µM both alone or in combination with entinostat (2.5 µM). TPCK is an NFκB inhibitor, U0126 and PD98059 are MEK1 and 2 inhibitors, SB203580 and SB202190 are p38 inhibitors and SP600125 is a JNK inhibitor.

Supplementary Figure S3

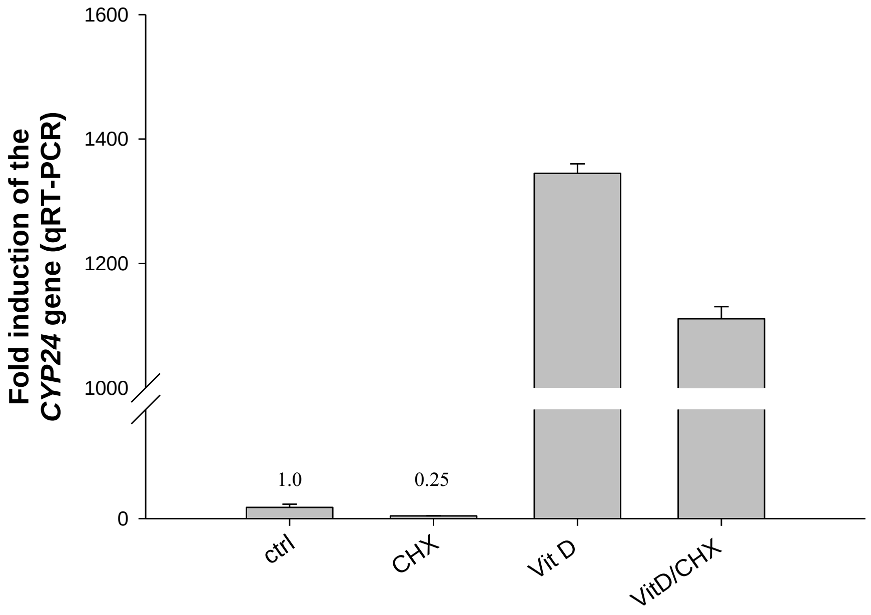

Supplementary Figure S3. Translational inhibition is not suppressing Vit D stimulated expression of the *CYP24* gene. *De novo* protein synthesis was inhibited in HT-29 cells by incubation with 1 µg/ml cycloheximide (CHX) in the presence or absence of 100 nM of Vit D for 24 h. The level of *CYP24* transcript was measured by qRT-PCR.
